# Quantitative ultrasound shear wave elastography (USWE)-measured tissue stiffness correlates with PIRADS scoring of MRI and Gleason score on whole-mount histopathology of prostate cancer: implications for ultrasound image-guided targeting approach

**DOI:** 10.1186/s13244-021-01039-w

**Published:** 2021-07-08

**Authors:** Wael Ageeli, Cheng Wei, Xinyu Zhang, Magdalena Szewcyk-Bieda, Jennifer Wilson, Chunhui Li, Ghulam Nabi

**Affiliations:** 1 Division of Imaging Sciences and Technology, School of Medicine, University of Dundee, Ninewells Hospital, Dundee, DD1 9SY UK; 2 Division of Population Health and Genomics, School of Medicine, University of Dundee, Dundee, DD1 9SY UK; 3 Department of Clinical Radiology, Ninewells Hospital, Dundee, DD1 9SY UK; 4 Department of Pathology, Ninewells Hospital, Dundee, DD1 9SY UK; 5 School of Science and Engineering, University of Dundee, Dundee, DD1 4HN UK; 6 Department of Radiological Sciences, Collage of Applied Medical Science, Jazan University, P.O Box 2128, Jazan, Saudi Arabia

**Keywords:** Prostate, Ultrasonography, Magnetic resonance imaging, Shear wave elastography

## Abstract

**Objective:**

To correlate quantitative tissue stiffness measurements obtained by transrectal ultrasound shear wave elastography (USWE) with PI-RADS scoring of multiparametric magnetic imaging resonance (mpMRI) using Gleason scores of radical prostatectomy as a reference standard.

**Patients and methods:**

196 men with localised prostate cancer were prospectively recruited into the study and had quantitative prostate tissue stiffness measurements in kilopascals (kPa) using transrectal USWE prior to radical prostatectomy. PI-RADS scores of mpMRI were also obtained in all the men. Imaging and histopathology of radical prostatectomy specimen were oriented to each other using patient specific customised 3D moulds to guide histopathology grossing of radical prostatectomy specimens. All included patients had confirmed PCa on TRUS-guided biopsies, had both USWE and mpMRI imaging data, and underwent radical prostatectomy. Chi-square test with 95% confidence interval was used to assess the difference between Gleason score (GS) of radical prostatectomy and PI-RADS classification, as well as GS of radical prostatectomy and stiffness (in Kpa) using USWE. The correlation coefficient (*r*) was calculated in order to investigate relation between PI-RADS classification and tissue stiffness in kPa.

**Results:**

There was a statistically significant correlation between USWE-measured tissue stiffness and GS (*χ*^2^ (2, *N* = 196) = 23.577, *p* < 0.001). Also, there was a statistically significant correlation between Gleason score and PI-RADS score (*χ*^2^ (2, *N* = 196) = 12.838, *p* = 0.002). High PIRADS on MRI and high stiffness on USWE (> 100 kPa) detected more than 80% and 90% high risk prostate cancer disease. However, a weak correlation coefficient of 0.231 was observed between PI-RADS score and level of tissue stiffness measured in kPa.

**Conclusion:**

Quantitative USWE and mpMRI using PI-RADS classification provide a good degree of prediction for Gleason score of clinically significant prostate cancer (csPCa). Stiffer lesions on ultrasound showed a weak correlation with PI-RADS scoring system. USWE could be used to target suspected prostate cancer.

## Key points

- The study confirms a strong correlation between PIRADS score on MRI and tissue stiffness measured by ultrasound shear wave elastography and Gleason score of histology in prostate cancer.
- The study used imaging-derived 3D printed moulds to orient histopathology section to pre-surgical imaging.
- Findings from this study have implications for image guided biopsies and image fusion technology for the detection of prostate cancer.

## Introduction

Prostate cancer (PCa) is one of the most common cancers responsible for mortality in men [1]. Screening of asymptomatic men for PCa is carried out using serum prostate-specific antigen (PSA) combined with digital rectal examination (DRE). The specificity of serum PSA test and DRE remains low [2–4]. Therefore, confirmation of diagnosis requires further microscopic examination of tissue obtained using transrectal or transperineal ultrasound-guided biopsies. Histopathology of prostate biopsy is necessary to confirm diagnosis.

There is an increased awareness that traditional transrectal ultrasound-guided systemic biopsy approach based on random sampling misses csPCa. This is due to limitations of B-mode greyscale ultrasound imaging which has a high false-negative rate [5, 6]. To mitigate this and improve sampling of prostate gland, increased number of systematic biopsies have been suggested which can result in associated increased risk of complications [7, 8]. Therefore, imaging facilitated sampling of abnormal areas is an emerging strategy to achieve a balance.

Multiparametric magnetic resonance imaging (mpMRI) and transrectal ultrasound (TRUS) are the primary imaging modalities for characterising PCa prior to biopsy. A grey-scale transrectal ultrasound (TRUS) is a low cost, but has limited sensitivity and specificity (40–50%) in the detection or characterisation of PCa [9, 10]. mpMRI has been promising in characterising abnormal areas using PI-RADS score, and studies have reported that MRI-TRUS fusion-guided targeted biopsy of the prostate has a higher detection rate of csPCa compared to systematic biopsy [11, 12]. On contrary, other studies were unable to demonstrate this superiority of MRI-TRUS fusion-guided targeted biopsy in the detection of PCa compared to systematic biopsy [13, 14]. mpMRI has limitations such as contraindications (Pacemaker, claustrophobia, etc.), scan time, and high cost [15].

We and others have recently reported on quantitative transrectal ultrasound shear wave elastography in the detection and characterisation of prostate cancer [16–18]. USWE-measured tissue stiffness can be a good biophysical marker of prostate Gleason score [16]; however, we do not know, how this will compare to mpMRI-based PI-RADS scoring. If further research validates good correlation then USWE-detected lesions could be targeted as an alternate technique to MRI-TRUS fusion-guided method. Moreover, stiffer tissues provide a unique microenvironment to cancer cells and promote metastases [17] and quantitative measurement may help in stratifying men to different therapeutic options. It is well known that cells respond and change their biophysical characteristics in response to cues from the extracellular matrix [17]. Whether measurement and quantification of tissue stiffness using ultrasound can provide some insight into tumour behaviour in prostate cancer has been scantly reported [18].

Quantitative image analysis for size, texture, and number of tumours is used clinically to assess tumour response to therapeutic interventions [19]. However, reproducible, easy to interpret, and quantifiable imaging modality in prostate cancer detection is still not clinically available. USWE with pseudo-colour-coded quantifiable images into red and blue provides an easy estimate of tissue stiffness (Fig. 2b). The aim of the present study was to find out the correlation between USWE-measured stiffness and PI-RADS scores using mpMRI in detecting risk-stratified Gleason scores of prostate cancer.

## Patients and methods

### Study design and patients

The study assessed images retrospectively obtained as part of protocol-driven study with prior ethical approval through East of Scotland Ethical committee and Caldicott permission (IGTCAL5626) to access the healthcare follow-up data [16]. Table 1 shows detailed patient and imaging characteristics.

**Table 1:** Patient and imaging characteristics

Patient characteristics	
No. pts	196
Age (years)	
Mean ± SD	66 ± 5
Median (IQR)	66.5 (63–72)
PSA level (ng/ml)	
Mean ± SD	12 ± 7.8
Median (IQR)	9.8 (7.5–13.1)
Prostate weight	
Mean ± SD	66 ± 29.7
Median (IQR)	59 (46.5–76)
PSA density (ng/ml^2^)	
Mean ± SD	0 ± 0.2
Median (IQR)	0.2 (0.1–0.2)

Stiffness measurement using USWE in Kilopascals (mean ± SD)	No. (%)
≤ 100 (94 ± 10.3)	43 (21.9)
100–130 (115 ± 11.4)	47 (24)
> 130 (138.4 ± 29)	106 (54.1)

Gleason score	No. (%)
3 + 3	3 (1.5)
3 + 4	94 (48)
4 + 3	33 (16.8)
3 + 5	18 (9.2)
4 + 4	3 (1.5)
4 + 5 or greater	45 (23)

PI-RADS categories	No. (%)
PIRADS 3	16 (8.16)
PIRADS 4	45 (22.96)
PIRADS 5	135 (68.88)

Inclusion criteria were:

1. Patients with confirmed PCa on TRUS-guided biopsies, coupled with both availability of both pre-surgical USWE and mpMRI, and
2. The diagnosis confirmed by radical prostatectomy as a gold standard.

**Fig. 1:**
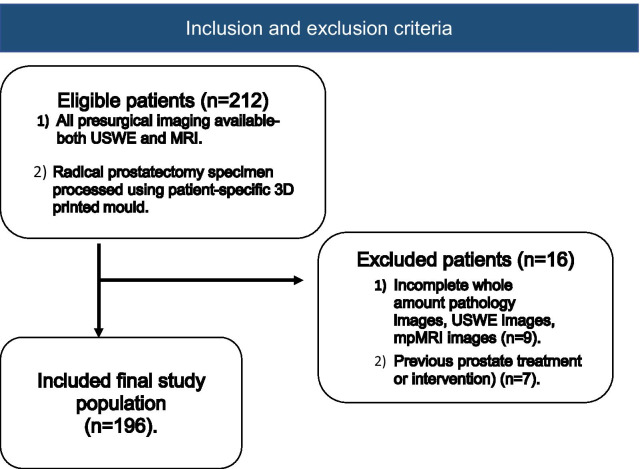
Flowchart shows inclusion and exclusion criteria and the patients selection process

Patients were excluded if whole amount pathology images, USWE images, mpMRI images were unavailable (*n* = 16) or patients with prior radiotherapy, trans-urethral resection of prostate and hormonal therapy. a total of 196 patients, met the inclusion criteria (Fig. 1).

The primary outcome of the study was the degree of correlation between USWE-measured tissue stiffness and PI-RADS scores of mpMRI using histopathology of radical prostatectomy as a reference standard. The secondary outcome was the correlation between PI-RADS score and GS of histopathology.

### Multiparametric magnetic resonance imaging

MRI imaging was conducted for each patient 6–8 weeks after biopsy confirmed prostate cancer with a 3-T scanner (TIM Trio, Siemens, Erlangen, Germany) to avoid blood atrifacts caused by biopsy. MRI protocol for prostate cancer was acquired from the 2012 European Society of Uro-radiology Guidelines (ESUR)[20]. T1-weighted imaging (T1WI), T2-weighted imaging (T2WI), diffusion-weighted imaging (DWI); apparent diffusion coefficient (ADC), and dynamic contrast enhancement (DCE) were carried out with a transabdominal external phased array coil. Table 2 summarises the MRI acquisition protocol. All MRI images were analysed by two experienced uro-radiologists (SMB, SJ) working in consensus and blinded to clinicopathological features. The lesions seen on mpMRI were classified using PI-RADS v2 scoring system.

**Table 2:** MRI acquisition parameters

	T1WI		High resolution T2WI		DWI		DCE
	Axial	Sagittal	Axial	Coronal	DWI	DWI high b-value	Dyn Gd-MRI
TR (ms)	650	6000	4000	5000	3300	3300	4.76
Sequence	2DTSE	2DTSE	2DTSE	2DTSE	2DEPI	2DEPI	3D VIBE
TE (ms)	11	102	100	100	95	95	2.45
Flip angle (°)	150	140	150	150	–	–	10
Slice thickness (mm)	3	3	3	3	3	3	3
Slice gap (mm)	0.6	0.6	0.6	0.6	0	0	0.6
Resolution (pixels)	320	320	320	320	192	192	192
FOV (mm)	200	200	200	200	280	280	280
b-values (s/mm^2^)	–	–	–	–	50, 100, 500, 1000	2000	–
Temporal resolution (s)	–	–	–	–	–	–	–

### Ultrasound shear wave elastography

**Fig. 2:**
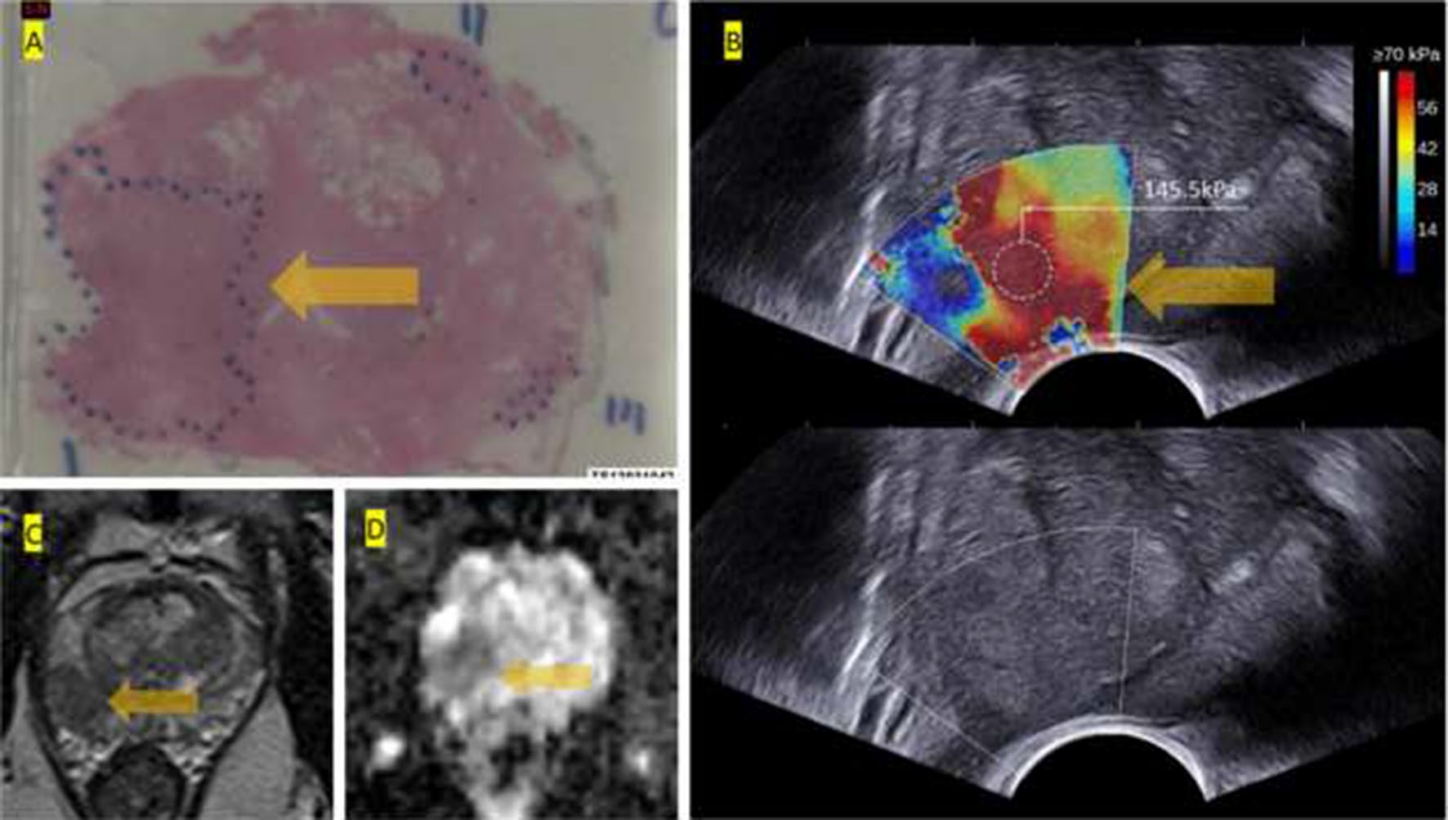
**a** Histopathology of whole-mount prostate with Gleason score 4 + 5 cancer in dotted area. **b** Ultrasound shear wave elastography USWE with pseudo-colour map; note cursor in red area with bar showing a quantitative stiffness measurement of 145.5 kPa (very high grade). B-Mode ultrasound showing no clue of suspected cancer. **c**, **d** T2w and ADC map images of the lesion from 3 T mpMRI scored as PI-RADS 5

The USWE technique has been described in detail in previous publications [21, 22]. All USWE images were obtained using a transrectal endocavitory transducer (SuperSonic Imagine, Aix en Provence, France) with patients either in lateral or lithotomy position. USWE mode was applied and elastograms of the prostate were acquired from cranial to caudal direction for each prostate lobe (Fig. 2). USWE examinations were performed by an experienced urologist with more than 10 years of experience in transrectal ultrasound. The USWE images were taken from base to apex in transverse planes with a gap of 4–6 mm. The most suspect planes containing cancer were labelled and rebuilt offline into 3D images. Rotating transducer in different directions to scan suspicious cancer regions ensured verification of abnormalities and accurate measurement of their dimensions. The ratio between abnormal and normal areas and three stiffness measurements of shear wave speed in m/s or Young’s modulus in kPa using pseudo-colour map with a 82.6 kPa as a cutoff value for benign vs. malignant tissue from previous published paper was recorded by three researchers (G.N., C.W. and D.U.) independently [23]. Based on data from same study, we categorised tissue stiffness into < 100 kPa; 100–130 kPa; and > 130 kPa.

### Radical prostatectomy histopathology as reference standard

**Fig. 3:**
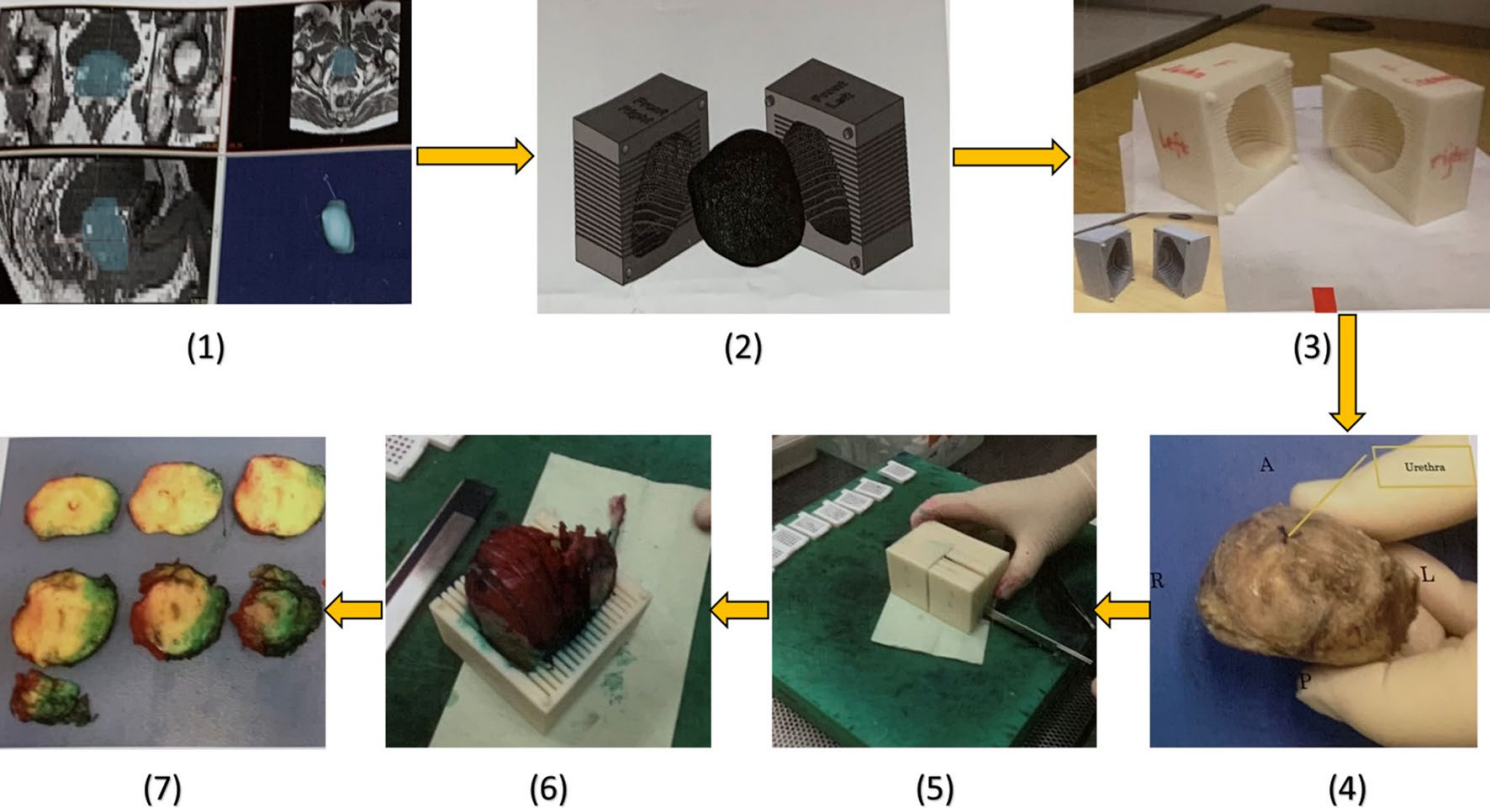
3D customised mould steps: (**1**) segmentation of MRI data in biomedical software MIMICS; (**2**) mold making in CAD software SolidWorks; (**3**) Ed printout from rapid prototyping machine Makerbot; (**4**) post-radical prostatectomy specimen before dyeing and placing in the mold; (**5**) slicing of the prostate specimen with single blade; (**6**) the tissue slices arranged from the apex to the base; (**7**) the tissue slices are arranged from the apex to the base

Patient-specific customised 3D moulds were built using imaging and 3-D printed according to our published protocol for each included men in the study [24]. The patient-specific customised 3D moulds were printed before surgery based on the T2-weighted mpMRI prostate images. They were built to keep prostates after surgical removal in the same form and orientation as seen on MRI. The 3D mould contains a series of evenly spaced parallel slits, each corresponding to a recognised slice of T2-weighted MRI (Fig. 3). This allows pathologist to gross specimen in the same orientation as imaging. The prostate specimens were immediately cut from base to apex in the axial orientation using a multi-bladed cutting tool [24].

### Data analysis

In all patients, pathological GS was determined by an experienced uro-pathologist (J.W.). Chi-square test was conducted to evaluate the difference between GS of radical prostatectomy and PI-RADS classification, as well as GS of radical prostatectomy and tissue stiffness (in kPa) using USWE. Pearson Chi-square value, degree of freedom, *p* value and 95% confidence intervals were calculated and presented. The degrees of freedom (df) for the Chi-square were calculated using the following formula: df = (*r* − 1)*(*c* −1) where r was the number of rows and c was the number of columns. The confidence interval (CI) calculated the lower and upper limits of the 95% confidence interval for the difference between two independent proportions, according to the method described by Robert Newcombe [25], derived from a procedure outlined by Wilson [26]. The CI calculation used the Wilson procedure with a correction for continuity.

In order to investigate the correlation between two categorical variables, Chi-square test of independence was applied on PI-RADS classification and stiffness data. Then the correlation coefficient (*r*) was calculated by the formula below, where *χ*^2^ was the Pearson Chi-square value and n equalled to the total number of patients with prostate cancer:

$$r = \sqrt {\frac{{\chi ^{2} }}{{\chi ^{2} + n}}}$$

The correlation coefficient greater than 0.5 suggested a moderate positive relationship between the two variables and the correlation coefficient in the range 0.7 ≤ *r* ≤ 1.0 represents a strong positive association.

In this study, Gleason scores 3 + 3 and 3 + 4 were considered as low/intermediate risk prostate cancer (group 1); Gleason score ≥ 4 + 3 was considered to be high-risk prostate cancer following University College London (UCL) 1 definition, (group 2) [27]. The data included in this study are available to third party through proper request as per institutional guidelines.

## Results

A total of 196 patients of clinically localised PCa with a mean age of 66 years (range 53–77 years), and mean PSA level of 11.7 ng/ml (range 0.1–47.7 ng/ml) were enrolled into the study. All patients had extraperitoneal laparoscopic radical prostatectomy and patient-specific 3D moulds were fabricated using MRI images. Of the 196 patients, 3 patients with Gleason score 6, 127 patients with Gleason score 7, and 66 with Gleason score ≥ 8 disease on radical prostatectomy histopathology.

Table 3 demonstrates the results of the Chi-square test of independence that examined the relation between those with low or intermediate risk disease or not and PI-RADS classification in patients with prostate cancer. The relation between these variables was statistically significant, *χ*^2^ (2, *N* = 196) = 12.838, *p* = 0.002. There was a difference in the distribution of patients with PI-RADS 3–5 between the low/intermediate risk GS group and the high-risk GS group. Approximately 80% of the patient in group 2 had a very high probability of csPCa (PI-RADS 5). Over 13% of the patients from group 2 had an intermediate probability of csPCa (PI-RADS 3).

**Table 3:** Distribution of PI-RADs classification using MRI and tissue stiffness using USWE in low/intermediate risk and high risk cancer prostate cancers based on Gleason score

Measurements	Low/intermediate risk^#^ GS group (*n* = 97)	%	High risk^^^ GS group (n = 99)	%	Pearson Chi-square	Degree of freedom	*P* value
PI-RADS classification*using MRI							
PI-RADS 3	13	13.4	3	3.0			
PI-RADS 4	28	28.9	17	17.2	12.838	2	0.002
PI-RADS 5	56	57.7	79	79.8			
Stiffness (in Kpa) using USWE							
< 100	33	34.0	10	10.1			
100–130	12	12.4	35	35.4	23.577	2	< 0.001
> 130	52	53.6	54	54.5			

*PI-RADS classification 3 means intermediate probability of clinically significant prostate cancer, 4 means high probability and 5 means very high probability of clinically significant prostate cancer; # Gleason score 3 + 3 and 3 + 4 were considered as low/intermediate risk cancer

Table 3 also shows the association between those with low/intermediate risk GS or not and tissue stiffness in kPa using USWE in patients with prostate cancer. The association between high risk GS or not and tissue stiffness in Kpa using USWE was statistically significant, *χ*^2^ (2, *N* = 196) = 23.577, *p* < 0.001. There was a difference in the distribution of measured stiffness between the low/intermediate GS group and the high risk GS group. About 90% of patients in the high risk GS group had a high stiffness (> 100 kPa) when using USWE. One third of the patients in the low/intermediate GS group was tested to have a low stiffness (< 100 kPa).

The association between PI-RADS and tissue stiffness by USWE is presented in Table 4. Chi-square test and weighted kappa of independency and agreement showed a weak relation between PI-RADS classification and tissue stiffness in patients with prostate cancer, *χ*^2^ (4, *N* = 196) = 11.084, *p* = 0.026. The correlation coefficient of the Chi-square, linear weighted kappa, and quadratic weighted kappa are 0.231, 0.034, and 0.078, respectively, which suggested association between PI-RADS by MRI and stiffness by USWE was weak.

**Table 4:** Correlation between tissue stiffness (in kPa) using USWE and PI-RADS classification using MRI in prostate cancer

PI-RADS classification*	Stiffness (in Kpa, *N* = 196)					%	Pearson Chi-square	Degree of freedom	*p* value	*r*	*κ* (linear weighted)	*κ* (quadratic weighted)	
	< 100 (*n* = 43)	%	100–130 (*n* = 47)	%	> 130 (*n* = 106)								
PI-RADS 3	4	9.3	2	4.3	10	9.4		11.084	0.026	0.231	0.034	0.078	
PI-RADS 4	17	39.5	7	14.9	21	19.8							
PI-RADS 5	22	51.2	38	80.8	75	70.8							

^*^PI-RADS classification 3 means intermediate probability of clinically significant prostate cancer, 4 means high probability of clinically significant prostate cancer and 5 means very high probability of clinically significant prostate cancer

## Discussion

Risk stratification of prostate cancer based on imaging features remains a major focus of research and present study cohort in a non-screened population of men with prostate cancer provided an opportunity to test transrectal USWE as an emerging imaging modality in comparison with mpMRI and histopathology of whole mount prostate gland. This is the largest series of cases reporting correlation of both mpMRI and USWE with the histopathological GS. Multiparametric MRI and USWE were both very promising imaging modalities in characterising and differentiating low/intermediate risk cancer from high risk disease based on Gleason Score (GS). The study used radical prostatectomy histopathology as a reference standard for GS to avoid under-reporting of high risk disease on biopsy. Patient-specific mould-based orientation between histopathology and imaging ensured a better comparison than reported before. Statistically significant correlations were observed between risks of Gleason score and PI-RADS classification on mpMRI. Similarly, a statistically significant high risk Gleason score on histopathology was seen in patients with a high tissue stiffness on USWE imaging. However, a trend was seen where higher PI-RADS score was associated with stiffer prostate cancer lesions with a weak correlation. These findings have implications for USWE-guided biopsy of prostate cancer as an alternate to a more commonly practiced MRI-US fusion technique; however, further research is required including external validity.

Slaoui H et al. in a retrospective analysis assessed correlation between PIRADS score and Gleason score of csPCa in 74 patients and showed no statistically significant correlation between PIRADS score and Gleason score [28]. This is in contrast to our study, where the correlation of the two groups was statistically significant. In an another study by Kızılay et al. [29], a statistically significant correlation was reported between PIRADS score and Gleason score similar to findings of our study. The authors used whole-mount histopathology in a case series of 177 men as a reference standard; however, no patient-specific moulds were used in contrast to what we have carried out in the present study.

Quantitative measurements of tissue stiffness using USWE have shown excellent characterisation and detection of csPCa [30–32]. This is based on a higher cellular density and microvascularisation with larger lesions producing more stromal reactions and collagen deposition causing stiffer and more aggressive tissue [10, 33, 34]. In previous reports utilising USWE, Woo et al. and Sunao et al. reported a significant association between tissue stiffness measurements and Gleason score of csPCa [33, 35], again similar to our findings.

USWE was able to provide additional information for detecting PCa and biopsy guidance and appeared to be comparable to other imaging modalities like MRI [12]. However, only a few studies reported a direct compression between MRI and USWE for PCa detection. One of these studies was done by Junker D et al. [36] who compared MRI with real-time elastography and reported that both modalities have a high sensitivity in detecting high-risk PCa. The small study of 39 men undergoing radical prostatectomy used real-time elastography which is significantly different from USWE described in the present study. Most importantly, no pressure is required by the operator in USWE in contrast to real-time elastography. In our study, we found that using the stiffness values and PI-RADS score to predict the Gleason score of PCa was statistically significant. Combining mpMRI and USWE could decrease under the detection rate of csPCa.

The current study has limitations. Firstly, the inclusion criteria used in this study allowed only men with known localised prostate cancer opting for radical surgery and this introduces a selection bias. Secondly, the study is from a single institution with experienced operator performing the test, most certainly USWE. Thirdly, the clinical utility of pre-biopsy imaging needs further testing in men with raised PSA and abnormal digital examination. Further research is also needed to assess its external validation. We have recently reported role of USWE in predicting upgrading of GS from biopsy to radical surgery histopathology and further research should focus on the external validity of this study in men suspected to have prostate cancer.

A significant number of studies report improved precision of biopsy sampling of prostate cancer including image fusion of MR/US [37, 38]. Targeting of csPCa using clinical imaging has potential of reducing over-detection and over-treatment of prostate cancer, an issue associated with random systemic biopsies. Aigner et al. [39] reported a significant improvement in prostate cancer detection rate per-core of biopsy tissue sample using real-time elastographic detection of prostate cancer. Findings from the present study should allow us to improve real-time targeting of csPCa using transrectal USWE and decrease number of biopsies with associated patient morbidity, in particular, when increasing numbers may not be yielding a higher detection rate. In a separate study [16], negative predictive value of transrectal USWE was 97% which certainly takes us closer to avoiding prostate biopsy in men wherever indicated.

## Conclusion

USWE and mpMRI are promising imaging modalities in detecting csPCa. The PI-RADS score of mpMRI and stiffness value of USWE can predict risk-based Gleason score and can be used to facilitate image-guided sampling of csPCa.

## Data Availability

The data are available for scrutiny from external requests.

## References

[1] Center MM, Jemal A, Lortet-Tieulent J (2012). International variation in prostate cancer incidence and mortality rates. Eur Urol.

[2] Ji Y, Litao R, Ren W (2019). Stiffness of prostate gland measured by transrectal Real-Time shear wave elastography for detection of prostate cancer: a feasibility study. Br J Radiol.

[3] Ziparo E, Petrungaro S, Marini ES (2013). Autophagy in prostate cancer and androgen suppression therapy. Int J Mol Sci.

[4] Heidenreich A, Bellmunt J, Bolla M (2011). EAU guidelines on prostate cancer, Part 1: Screening, diagnosis, and treatment of clinically localised disease. Eur Urol.

[5] Stroumbakis N, Cookson MS, Reuter VE, Fair WR (1997). Clinical significance of repeat sextant biopsies in prostate cancer patients. Urology.

[6] Neutel CI, Gao RN, Blood PA, Gaudette LA (2006). Trends in prostate cancer incidence, hospital utilization and surgical procedures, Canada, 1981–2000. Can J Public Heal.

[7] De La Taille A, Antiphon P, Salomon L (2003). Prospective evaluation of a 21-sample needle biopsy procedure designed to improve the prostate cancer detection rate. Urology.

[8] Eskew LA, Bare RL, McCullough DL (1997). Systematic 5 region prostate biopsy is superior to sextant method for diagnosing carcinoma of the prostate. J Urol.

[9] Fu S, Tang Y, Tan S, Zhao Y, Cui L (2020). Diagnostic value of transrectal shear wave elastography for prostate cancer detection in peripheral zone: comparison with magnetic resonance imaging. J Endourol.

[10] Barr RG, Cosgrove D, Brock M, Cantisani V, Correas JM, Postema AW (2017). WFUMB guidelines and recommendations on the clinical use of ultrasound elastography: part 5. Prostate. Ultrasound Med Biol.

[11] Porpiglia F, Manfredi M, Mele F (2017). Diagnostic pathway with multiparametric magnetic resonance imaging versus standard pathway: results from a randomized prospective study in biopsy-Naïve patients with suspected prostate cancer. Eur Urol.

[12] Schoots IG, Roobol MJ, Nieboer D (2015). Magnetic resonance imaging-targeted biopsy may enhance the diagnostic accuracy of significant prostate cancer detection compared to standard transrectal ultrasound-guided biopsy: a systematic review and meta-analysis. Eur Urol.

[13] Tonttila PP, Lantto J, Pääkkö E (2016). Prebiopsy multiparametric magnetic resonance imaging for prostate cancer diagnosis in biopsy-naive men with suspected prostate cancer based on elevated prostate-specific antigen values: results from a randomized prospective blinded controlled trial. Eur Urol.

[14] Baco E, Rud E, Eri LM (2016). A Randomized controlled trial to assess and compare the outcomes of two-core prostate biopsy guided by fused magnetic resonance and transrectal ultrasound images and traditional 12-core systematic biopsy. Eur Urol.

[15] Kesch C, Schütz V, Dieffenbacher S (2018). Multiparametric MRI fusion-guided biopsy for the diagnosis of prostate cancer. Curr Opin Urol.

[16] Wei C, Li C, Szewczyk-Bieda M (2018). Performance characteristics of transrectal shear wave elastography (SWE) imaging in the evaluation of clinically localised prostate cancer: a prospective study. J Urol.

[17] Yue X, Nguyen TD, Zellmer V, Zhang S, Zorlutuna P (2018). Stromal cell-laden 3D hydrogel microwell arrays as tumor microenvironment model for studying stiffness dependent stromal cell-cancer interactions. Biomaterials.

[18] Wei C, Zhang Y, Malik H (2019). Prediction of postprostatectomy biochemical recurrence using quantitative ultrasound shear wave elastography imaging. Front Oncol.

[19] Cai W-L, Hong G-B (2018). Quantitative image analysis for evaluation of tumor response in clinical oncology. Chronic Dis Transl Med.

[20] Barentsz JO, Richenberg J, Clements R (2012). ESUR prostate MR guidelines 2012. Eur Radiol.

[21] Bercoff J, Tanter M, Fink M (2004). Supersonic shear imaging: a new technique. IEEE Trans Ultrason Ferroelectr Freq Control.

[22] Bercoff J, Chaffai S, Tanter M (2003). In vivo breast tumor detection using transient elastography. Ultrasound Med Biol.

[23] Wei C, Li C, Szewczyk-Bieda M (2018). Performance characteristics of transrectal shear wave elastography imaging in the evaluation of clinically localized prostate cancer: a prospective study. J Urol.

[24] Sheikh N, Wei C, Szewczyk-Bieda M (2017). Combined T2 and diffusion-weighted MR imaging with template prostate biopsies in men suspected with prostate cancer but negative transrectal ultrasound-guided biopsies. World J Urol.

[25] Newcombe RG (1998). Interval estimation for the difference between independent proportions: comparison of eleven methods. Stat Med.

[26] Wilson EB (1927). Probable inference, the law of succession, and statistical inference. J Am Stat Assoc.

[27] Kanthabalan A, Abl-Azzeez M, Arya M (2016). Transperineal MRI-targeted biopsy versus transperineal template prostate mapping biopsy in the detection of localised radio-recurrent prostate cancer. Clin Oncol (R Coll Radiol).

[28] Slaoui H, Neuzillet Y, Ghoneim T (2017). Gleason score within prostate abnormal areas defined by multiparametric magnetic resonance imaging did not vary according to the pirads score. Urol Int.

[29] Kızılay F, Çelik S, Sözen S (2020). Correlation of Prostate-Imaging Reporting and Data Scoring System scoring on multiparametric prostate magnetic resonance imaging with histopathological factors in radical prostatectomy material in Turkish prostate cancer patients: a multicenter study of t. Prostate Int.

[30] Zhang M, Wang P, Yin B (2015). Transrectal shear wave elastography combined with transition zone biopsy for detecting prostate cancer. Natl J Androl.

[31] Jain MA, Sapra A. Cancer prostate screening. In: StatPearls. StatPearls Publishing; 2020. 32310541

[32] Muthigi A, George AK, Sidana A (2017). Missing the mark: prostate cancer upgrading by systematic biopsy over magnetic resonance imaging/transrectal ultrasound fusion biopsy. J Urol.

[33] Woo S, Suh CH, Kim SY, Cho JY, Kim SH (2017). Shear-wave elastography for detection of prostate cancer: a systematic review and diagnostic meta-analysis. AJR Am J Roentgenol.

[34] Shoji S, Hashimoto A, Nakamura T (2018). Novel application of three-dimensional shear wave elastography in the detection of clinically significant prostate cancer. Biomed Rep.

[35] Shoji S, Hashimoto A, Nakamura T (2018). Novel application of three-dimensional shear wave elastography in the detection of clinically significant prostate cancer. Biomed Rep.

[36] Junker D, De Zordo T, Quentin M (2014). Real-time elastography of the prostate. Biomed Res Int.

[37] Szewczyk-Bieda M, Wei C, Coll K (2019). A multicentre parallel-group randomised trial assessing multiparametric MRI characterisation and image-guided biopsy of prostate in men suspected of having prostate cancer: MULTIPROS study protocol. Trials.

[38] Alqahtani S, Wei C, Zhang Y (2020). prediction of prostate cancer Gleason score upgrading from biopsy to radical prostatectomy using pre-biopsy multiparametric MRi piRADS scoring system. Sci Rep.

[39] Aigner F, Pallwein L, Junker D (2010). Value of real-time elastography targeted biopsy for prostate cancer detection in men with prostate specific antigen 1.25 ng/ml or greater and 4.00 ng/ml or less. J Urol.

